# Under-25s show the clearest post-2021 rebound in mental health: HILDA 2001–2024

**DOI:** 10.1177/00048674261439582

**Published:** 2026-05-16

**Authors:** Sergey Alexeev, Nick Glozier

**Affiliations:** 1 Nura Gili: Centre for Indigenous Programs, Co-Design Health Research and Innovation Team, University of New South Wales, Sydney, NSW, Australia; 2 Central Clinical School, The University of Sydney, Sydney, NSW, Australia

## Introduction

Across countries, population mental health trends have become increasingly age-divergent: younger people show worsening outcomes while older adults remain comparatively stable, so aggregate averages can mask where change is concentrated (Krokstad et al., 2022). In Australia, repeated national surveys indicate rising psychological distress and increasing prevalence of common mental disorders, with the largest changes occurring among adolescents and young adults (Enticott et al., 2022; Slade et al., 2024). Longitudinal evidence from the Household, Income and Labour Dynamics in Australia (HILDA) Survey similarly points to a generational decline in mental health over the 21st century driven largely by younger cohorts (Botha et al., 2023). The COVID-19 period added an acute shock, with longitudinal syntheses documenting a deterioration in mental health in 2020, followed by heterogeneous trajectories thereafter (Robinson et al., 2022).

Using 24 HILDA waves (2001–2024), we provide a highly descriptive account of long-run trends in the 36-Item Short Form Survey (SF-36) mental health scale by age group and then focus on the post-2021 period (2021–2024) to quantify recovery among young people. We then test whether that rebound appears concentrated in more advantaged areas or instead broadly shared across area-level socio-economic contexts, using three 2021 Socio-Economic Indexes for Areas (SEIFA) measures chosen for interpretability and consistent availability across waves.

## Methods

We analysed annual data from the HILDA Survey, a nationally representative household panel study (Summerfield et al., 2025; Watson and Wooden, 2012). Mental health was measured using the SF-36 mental health subscale (0–100; higher indicates better mental health) (Sanson-Fisher and Perkins, 1998; Ware and Sherbourne, 1992). We constructed a person-year dataset (one record per respondent per wave), recoding HILDA negative non-response codes to missing.

**Figure 1.**
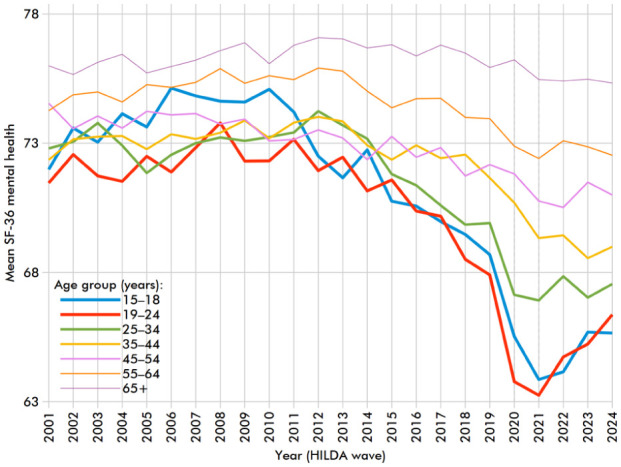
SF-36 mental health by age group, Australia. Source: HILDA Survey 2001–2024. Lines show the survey-weighted mean SF-36 mental health scores (0–100; higher scores indicate better mental health) by year for seven age groups. Respondents with missing mental health, missing age or non-positive weights are excluded.

In Figure 1, we estimated survey-weighted mean SF-36 mental health by year within seven age groups using the responding person population weights (hhwtrp).

In Table 1, we focused on youth aged 15 to 24 years observed during 2021–2024 and defined a linear post-2021 trend (Trend = 0 in 2021, 1 in 2022, 2 in 2023, 3 in 2024). We related mental health to three SEIFA 2021 indices of area socio-economic context: the Index of Relative Socio-economic Advantage and Disadvantage (IRSAD), the Index of Economic Resources (IER) and the Index of Education and Occupation (IEO), each entered as a z-score and analysed one at a time for interpretability. For each index, we estimated:

$$\begin{array}{l} \mathrm{ghmhit}=\alpha \;\;+\;\beta_{1}{\mathrm{Trend}}_{t}+\;\beta_{2}{\mathrm{Index}}_{it}\\ \;\;+\;\beta_{3}({\mathrm{Trend}}_{t}\times {\mathrm{Index}}_{it})\\ \;\;+\;\gamma_{1}{\mathrm{Female}}_{it}+\;\gamma_{2}{\mathrm{Age}}_{it}+\varepsilon_{it}. \end{array}$$

We used the Taylor linearised survey inference with svyset (strata xhhstrat, PSU xhhraid) and the Self-Completion Questionnaire responding person population weight (hhwtsc), and we estimated the youth domain using subpop so that variance estimation used the full 2021–2024 design sample.

**Table 1.** Youth mental health rebound by socio-economic context.

	(1)	(2)	(3)
Outcome	SF-36 mental health		
Index	IRSAD	IER	IEO
Trend (T)	0.718***	0.735***	0.709***
	(0.231)	(0.231)	(0.230)
	0.002	0.002	0.002
Index (I)	1.003*	1.544**	0.419
	(0.592)	(0.680)	(0.519)
	0.090	0.023	0.420
T × I	0.026	–0.009	0.158
	(0.265)	(0.315)	(0.232)
	0.920	0.978	0.495
Female	–6.537***	–6.439***	–6.563***
	(0.687)	(0.687)	(0.685)
	< 0.001	< 0.001	< 0.001
Age	–0.028	0.033	–0.045
	(0.118)	(0.116)	(0.119)
	0.809	0.779	0.706
Constant	67.507***	66.157***	67.916***
	(2.372)	(2.338)	(2.421)
	< 0.001	< 0.001	< 0.001
Youth *N* (unweighted)	8193		
Youth pop. size (weighted)	13,217,909		
All obs (design)	65,668		

Source: HILDA Survey 2021–2024.

Survey-weighted regressions of SF-36 mental health on a fully interacted Trend (2021–2024; 0–3) and SEIFA 2021 index (*z*-score), controlling for sex and age. IRSAD, IER and IEO denote SEIFA indices for advantage-disadvantage, economic resources and education/occupation. Near-zero Trend × Index interactions are consistent with a broad-based post-2021 rebound. Standard errors are in parentheses, and p-values are shown on the line below the corresponding standard error. *p < 0.10; **p < 0.05; *** *p* < 0.01.

## Results

Figure 1 shows a stable age gradient in SF-36 mental health over 2001–2024, with younger groups generally reporting lower mental health than older adults. The pandemic-era disruption is concentrated among adolescents and young adults: mean mental health declined sharply from 2019 to 2021 and then partially recovered by 2024. Among those aged 15–18 years, mean SF-36 mental health fell from 68.7 (2019) to 63.9 (2021) and rose to 65.7 in 2024 (+1.8 points from 2021), remaining 3.0 points below 2019. Among those aged 19–24 years, mean mental health declined from 67.9 (2019) to 63.2 (2021) and recovered to 66.4 in 2024 (+3.1 points from 2021), remaining 1.5 points below 2019. Changes in older age groups were smaller.

In pooled youth models for 2021–2024 (Table 1), the estimated recovery is consistent across socio-economic status (SES) specifications: mental health increased by around 0.71–0.74 points per year (all *p* = 0.002), implying an average gain of approximately 2.1–2.2 points between 2021 and 2024. Higher area SES was associated with modestly higher mental health levels in 2021 (significant for IER: 1 SD higher IER → 1.54 points higher mental health; *p* = 0.023), but the Trend × Index interactions were near zero and not statistically significant (*p* ⩾ 0.495), indicating no clear evidence that post-2021 recovery differed by area SES. Within the limits of these area-level measures, this suggests that the post-2021 improvement was broad-based rather than confined to more socio-economically advantaged youth. Across all models, young women reported substantially lower mental health than young men (approximately 6.4–6.6 points; *p* < 0.001).

## Discussion

Using two decades of nationally representative panel data, we show that the 2020–2021 deterioration in mental health was largest among adolescents and young adults and that recovery by 2024 was partial, leaving youth mental health below pre-pandemic levels. This pattern aligns with broader evidence that recent mental health change is concentrated in younger cohorts (Botha et al., 2023) and with national survey evidence of heightened mental health burden among young Australians in 2020–2022 (Slade et al., 2024).

Our post-2021 models suggest that the recovery slope from 2021 to 2024 was broadly shared across socio-economic contexts: while youth in more advantaged areas reported somewhat higher levels of mental health (particularly for economic resources), we found no evidence that the rate of improvement differed systematically by SEIFA 2021 indices. This complements longitudinal syntheses indicating heterogeneous but often partially improving trajectories after the initial COVID-era shock (Robinson et al., 2022).

This research letter is descriptive, and several limitations should be noted. First, the SF-36 mental health scale is self-reported and captures symptoms rather than clinical diagnoses (Ware and Sherbourne, 1992). Second, SEIFA indices are area-based and may not capture individual socio-economic circumstances. Third, we summarised the 2021–2024 change with a linear trend; non-linear recovery patterns may be missed. Finally, attrition and item non-response may shape observed trends, although we used HILDA weights and design variables to support population-representative inference (Summerfield et al., 2025). Despite these limitations, the concentration of decline and incomplete recovery among young people underscores the importance of ongoing surveillance and youth-focused prevention and service responses.
